# Viral CTL Escape Mutants Are Generated in Lymph Nodes and Subsequently Become Fixed in Plasma and Rectal Mucosa during Acute SIV Infection of Macaques

**DOI:** 10.1371/journal.ppat.1002048

**Published:** 2011-05-19

**Authors:** Thomas H. Vanderford, Chelsea Bleckwehl, Jessica C. Engram, Richard M. Dunham, Nichole R. Klatt, Mark B. Feinberg, David A. Garber, Michael R. Betts, Guido Silvestri

**Affiliations:** 1 Yerkes National Primate Research Center, Emory University, Atlanta, Georgia, United States of America; 2 Department of Pathology and Laboratory Medicine, University of Pennsylvania School of Medicine, Philadelphia, Pennsylvania, United States of America; 3 Merck Vaccines and Infectious Diseases, Merck and Co., Inc., West Point, Pennsylvania, United States of America; 4 Department of Microbiology, University of Pennsylvania School of Medicine, Philadelphia, Pennsylvania, United States of America; NIH/NIAID, United States of America

## Abstract

SIV_mac239_ infection of rhesus macaques (RMs) results in AIDS despite the generation of a strong antiviral cytotoxic T lymphocyte (CTL) response, possibly due to the emergence of viral escape mutants that prevent recognition of infected cells by CTLs. To determine the anatomic origin of these SIV mutants, we longitudinally assessed the presence of CTL escape variants in two MamuA*01-restricted immunodominant epitopes (Tat-SL8 and Gag-CM9) in the plasma, PBMCs, lymph nodes (LN), and rectal biopsies (RB) of fifteen SIV_mac239_-infected RMs. As expected, Gag-CM9 did not exhibit signs of escape before day 84 post infection. In contrast, Tat-SL8 escape mutants were apparent in all tissues by day 14 post infection. Interestingly LNs and plasma exhibited the highest level of escape at day 14 and day 28 post infection, respectively, with the rate of escape in the RB remaining lower throughout the acute infection. The possibility that CTL escape occurs in LNs before RBs is confirmed by the observation that the specific mutants found at high frequency in LNs at day 14 post infection became dominant at day 28 post infection in plasma, PBMC, and RB. Finally, the frequency of escape mutants in plasma at day 28 post infection correlated strongly with the level Tat-SL8-specific CD8 T cells in the LN and PBMC at day 14 post infection. These results indicate that LNs represent the primary source of CTL escape mutants during the acute phase of SIV_mac239_ infection, suggesting that LNs are the main anatomic sites of virus replication and/or the tissues in which CTL pressure is most effective in selecting SIV escape variants.

## Author Summary

Strong antiviral CD8+ T lymphocytes limit SIV replication by recognizing short pathogen-derived peptide epitopes. The cytotoxic CD8+ T cell responses specific for this highly mutable virus can select for viruses bearing mutations that prevent CD8+ T cell recognition of cells infected with these escape mutants. To determine the anatomic origin of these escape mutants, we tracked a particular escape mutant in multiple tissues (plasma virus, lymph nodes, rectal mucosa, and peripheral blood immune cells) during the early, acute phase of SIVmac239 infection of rhesus macaques. We found that escape mutants first reach high frequency in lymph nodes 2 weeks after infection, and the particular mutants generated in lymph nodes disseminate to other tissues by week 4. Furthermore, we found that epitope-specific CD8+ T lymphocyte responses in the lymph nodes and peripheral blood, but not the gut mucosa, are significantly correlated with the frequency of escape mutants in the plasma virus at week 4. This suggests that lymph nodes, and not the gut, are the primary site of anti-SIV CD8+ T cell responses and/or SIV replication during the acute phase of infection.

## Introduction

Human immunodeficiency virus (HIV) infection of humans and Simian Immunodeficiency Virus (SIV) infection of rhesus macaques (*Macaca mulatta*, RM) results in a progressive and irreversible decline of immune function characterized by depletion of CD4 T cells, chronic immune activation, and high susceptibility to opportunistic infections that is commonly referred to as AIDS.

While the host immune system mounts strong cellular and humoral immune responses against HIV and SIV, these responses ultimately fail to control virus replication in the overwhelming majority of infected individuals. A key reason underlying this immune failure is the extreme genetic variability of these primate lentiviruses, which occurs as a result of a high mutation rate caused by the relative infidelity of the HIV and SIV reverse transcriptases [1]. This extreme genetic diversity combined with a large *in vivo* effective population size [2] practically ensures that the virus will always be able to evade or “escape” from recognition by the host immune system. In clinical terms, these biological features of HIV in the absence of “natural immunity” against the virus are key indicators of the complexity and difficulty that the scientific community faces when trying to design an effective AIDS vaccine.

In the absence of immunogens that are able to predictably elicit the production of broadly reactive HIV- or SIV-specific neutralizing antibodies [3], [4], there has been significant interest in immunogens that elicit strong antiviral CD8+ T cell-mediated cytotoxic T lymphocyte (CTL) responses [5]. A large body of evidence indicates that CD8+ T cells do play a significant role in the control of HIV and SIV replication that involves both cytolytic and non-cytolytic mechanisms. First, CD8+ T cells can inhibit HIV and SIV replication in vitro [6], [7]. Second, there is a temporal association between post-peak decline of acute viremia and emergence of CD8+ T cell responses [8], [9]. Third, antibody-mediated *in vivo* depletion of CD8+ lymphocytes is consistently associated with increased virus replication in SIV-infected RMs [10], [11], [12]. Fourth, there is a strong association between specific major histocompatibility complex (MHC) alleles and ability to control virus replication during HIV and SIV infection [13]. In this context, the fact that CTL escape mutants consistently arise during both acute and chronic HIV/SIV infections [13] demonstrate the presence of selective CD8+ T cell-mediated immune pressure on the virus population. On the other hand, the fact that escape mutants are consistently observed is also an indicator of the overall inability of these cells to fully suppress virus replication.

SIV_mac239_ infection of RMs bearing the MamuA*01 MHC class I allele elicits CD8+ T cell responses against two very well characterized immunodominant epitopes: Tat-SL8 [14] and Gag-CM9 [15]. While SL8- and CM9-specific CD8+ T cell responses are both generated during the acute phase of infection [14], [16], the emergence of CTL escape mutants occurs much more rapidly in the Tat-SL8 epitope than it does in Gag-CM9 [14], [17], [18], presumably due to strong functional constraints imposed on the *gag* gene and the need for extra-epitopic, secondary compensatory mutations to allow effective virus replication [19], [20], [21]. Of note, in these studies the kinetics of the generation and fixation of CTL escape mutations occurring in the SL8 and CM9 regions of SIV was analyzed only in plasma virus, which is thought to provide an overall representation of the virus that are replicating within the host. However, to the best of our knowledge, a comprehensive and comparative longitudinal analysis of CTL escape mutants in the mucosal and lymphoid tissues of SIV-infected RMs has not been yet been conducted. Determining if CTL escape mutants emerge more rapidly in lymphoid or, alternatively, mucosal tissues would provide important information with respect to the predominant sites of CD8+ T cell-mediated immunological pressure in vivo.

In this study, we characterized the appearance, dynamics, and dissemination of CD8+ T cell escape mutants in lymphoid vs mucosal tissues during SIV_mac239_ infection of RMs. To this end, we conducted an extensive longitudinal assessment of viral sequences derived from plasma viral RNA as well as cell-associated viral DNA in peripheral blood mononuclear cells (PBMC), lymph node biopsies (LN), and biopsies of the rectal mucosa (RB) of 15 SIV-infected RMs. These animals were included in a previously published study designed to investigate the immunogenicity and protection from SIV_mac239_ challenge conferred by two MVA-base candidate AIDS vaccines expressing SIVmac *gag* and *tat* [22]. As expected, we observed that CTL escape mutants occurred early in the Tat-SL8 epitope and much later in the Gag-CM9 epitope. Importantly, we found that in all RMs, Tat-SL8 escape mutants appeared earlier and in higher frequency in LNs then in RBs, with variants found at high frequency in LNs at day 14 post infection becoming dominant in the RBs at day 28 post infection. These results indicate that LNs represent the primary source of CTL escape mutants during the acute phase of SIV_mac239_ infection, suggesting that LNs are the main anatomic sites of virus replication and/or the tissues in which CTL pressure is most effective in selecting SIV escape variants.

## Results

### Experimental design: SIV_mac239_ infection of 10 MVA-SIVgag/tat-vaccinated and 5 unvaccinated rhesus macaques

A group of fifteen MamuA*01-positive rhesus macaques (*Macaca mulatta*; RM) were infected intravenously with 10,000 TCID50 of SIV_mac239_ as part of a previous study designed to assess the immunogenicity and potential protection from challenge conferred by MVA-based candidate AIDS vaccines [22]. In this study, ten RMs were immunized three times with either MVA “wild-type” (5 animals) or a genetically engineered MVA in which the gene for the Uracil DNA Glycosidase (UDG) was deleted, i.e. ΔUDG (5 animals), with both vectors expressing the Gag and Tat proteins of SIV_mac239_. Five additional unvaccinated RMs were used as controls. In all animals, SIV-specific CD8+ T cell responses were measured at various time points post-immunization and post-challenge in multiple tissues, including PBMC, RB, and LN by tetramer staining for the Gag-CM9 and Tat-SL8 epitopes of SIV_mac239_. The main results of this study are summarized in Table S1. Briefly, administration of MVA-SIV immunogens resulted in a partial (∼1 log) and transient (60–120 days) decline in plasma viral load that did not translate into protection from CD4+ T cell depletion and disease progression. Of note, in four RMs the level of virus replication decreased to near undetectable levels during the chronic phase of infection, suggesting that these “controller” animals were able to mount antiviral immune responses that could successfully suppress virus replication *in vivo*. The large amount of virologic and immunologic data collected in this study combined with an extensive archive of tissue samples presented an ideal opportunity to explore in detail the relationship between SIV-specific CD8+ T cell responses in various tissues, and the appearance and dissemination of CTL escape mutants in a relatively large cohort of SIV_mac239_-infected RMs. Due to relatively modest protective effect of the used immunization regimen [22], we chose to conduct our analysis in the entire group of 15 SIV-infected animals without dividing them into vaccinated and controls.

To characterize the emergence of CTL escape mutants in our cohort of SIV_mac239_-infected RMs, we amplified a 435 nucleotide region surrounding the Gag-CM9 epitope and a 390 nucleotide region surrounding the Tat-SL8 epitope from reverse transcribed plasma viral RNA and genomic DNA derived from PBMCs, RB, and LNs collected at multiple time points post infection. These amplicons were then sequenced from one end using Roche's 454 pyrosequencing technology. After eliminating all sequence reads that did not meet the minimum set of quality criteria (see Materials and Methods), source animals for each read were identified via pre-determined barcodes, and the reads were aligned with the corresponding wildtype SIV_mac239_ sequence. High frequency insertions and deletions (indels) that resembled well-characterized artifacts of the 454 pyrosequencing procedure were repaired with reference to the wildtype SIV_mac239_ sequence. All resulting sequences that contained no indels and were at least 243 nucleotides long for Gag-CM9 and 220 nucleotides long for Tat-SL8 were included in all subsequent analyses. To ensure that the sequence selection process did not bias our results, all analyses were performed on the full set of reads that had been repaired indiscriminately using the SIV_mac239_ wildtype sequence. No bias was found in our results (data not shown).

### Escape in Gag-CM9 occurred in only two RMs during the chronic phase of infection via mutation of a single anchor residue

**Figure 1:**
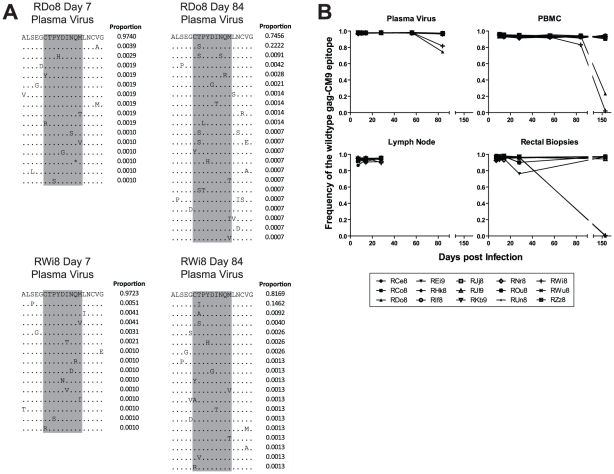
The gag-CM9 MamuA*01-restricted SIVmac239 CD8 T cell epitope escaped via a mutation of a single anchor residue that was observed late during the chronic phase of infection. (A) Four example amino acid alignments of the gag-CM9 epitope (indicated in grey) showing the changes in plasma viral populations between a baseline time point (day 7 post infection) and a time point in which escape is evident (day 84 post infection) in a two animals (RDo8 and RWi8). The proportion of reads bearing the sequence shown is indicated to the right of each line in the alignment. (B) The level escape at gag-CM9, expressed as the frequency of reads bearing the wildtype epitope in plasma virus, PBMC, LN and RB.

HIV and SIV *gag* genes are highly conserved and thus escape mutations in CD8+ T cell epitopes in this region are typically associated with either highly unfit viruses or the appearance of compensatory mutations outside of the epitope itself [20]. In order to characterize the rate and mechanisms of escape in the highly conserved Mamu-A*01-restricted Gag-CM9 epitope in our SIV_mac239_ infected RMs, we amplified and sequenced this epitope from plasma virus and cell-associated viral DNA in PBMCs, LN, and RB. Escape in Gag-CM9 occurred via mutations at the second position (threonine) in the epitope (Figure 1A). The substituted amino acids observed included serine, isoleucine, and cysteine. Interestingly, the threonine to cysteine amino acid substitution is achieved via a nucleotide substitution at the first and second positions of the threonine codon and was observed in the RB but not other tissues of a single animal, RDo8 (data not shown). There was no evidence of a significant increase in the frequency of the intermediate codons, thus suggesting that viruses bearing the resultant amino acid substitutions are at a severe selective disadvantage. However, it is also possible that the frequency of sampling during the chronic phase of SIV_mac239_ infection was not sufficient to detect the presence of relatively transient intermediate amino acid substitutions. As expected based on previous studies [17], [18], [20], escape in Gag-CM9 was observed in only two RMs (RDo8 and RWi8) and not until day 84 post infection, when escape mutants appeared in plasma virus and, although at lower frequencies, in PBMC-derived cell-associated virus (Figure 1B). In these two animals viruses isolated from RBs at day 168 post infection were almost entirely comprised of Gag-CM9 escape mutants (Figure 1B).

### Escape in Tat-SL8 occurred rapidly in plasma virus via multiple intra-epitopic amino acid substitutions

Previous studies of the kinetics of emergence of CTL escape in the Mamu-A*01-restricted Tat-SL8 immunodominant epitope have shown that in plasma virus, Tat-SL8 escape occurs during the early stages of SIV infection and via multiple amino acid substitutions [14], [23], [24]. For this reason, we focused our analysis of the emergence of Tat-SL8 escape mutants in our group of SIV_mac239_-infected RMs during the acute phase of infection. As described for the Gag-CM9 epitope, we PCR amplified the viral genomic region surrounding the Tat-SL8 epitope from reverse transcribed plasma viral RNA and sequenced it by 454 pyrosequencing.

**Figure 2:**
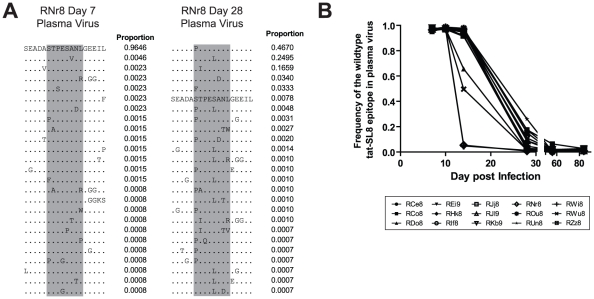
The tat-SL8 MamuA*01-restricted SIVmac239 CD8 T cell epitope escaped rapidly in the plasma virus via multiple amino acid substitutions during the acute phase of infection. (A) Two example amino acid alignments of the tat-SL8 epitope (indicated in grey) showing the changes in plasma viral populations between a baseline time point (day 7) and a time point in which escape is evident (day 28) in a single animal (RNr8). The proportion of reads bearing the sequence shown is indicated to the right of each line in the alignment. (B) The level escape at tat-SL8 in plasma virus expressed as the frequency of reads bearing the wildtype epitope during the first 84 days post infection.

As expected based on previous studies [14], [23], [24], the emergence of Tat-SL8 escape mutants consistently occurred at high frequency during acute SIV infection. In particular, we observed that CTL escape in Tat-SL8 occurred through one or more of several amino acid substitutions: serine to proline or phenylalanine at position 1, threonine to isoleucine at position 2, serine to leucine at position 5, and alanine to aspartic acid at position 6 (Figure 2A). These amino acid substitutions occur through single nucleotide mutations and have all been characterized as escape mutations in previous studies [14], [23], [24]. As shown in Figure 2B, Tat-SL8 escape mutants began to emerge by day 14 post infection in most RMs, with especially high levels of escape mutants evident in 4 animals (RDo8, ROu8, RWi8, RWu8). By day 28 post infection, the majority of plasma virus in all SIV-infected RMs was comprised of Tat-SL8 escape mutants, with the median frequency of the wildtype Tat-SL8 epitope at 0.08 (range, 0.008–0.261; Figure 2B). The frequency of the viruses bearing the wildtype Tat-SL8 epitope continued to decrease through day 84 post infection (mean, 0.01; range, 0.004–0.027). Overall, these data confirm the early emergence of numerous Tat-SL8 escape mutants, a finding that reflects both the selective pressure exerted by Tat-SL8-specific CD8+ T cell responses and the relative genetic flexibility of Tat in maintaining its function despite the presence of these mutations [19] .

### Lymph nodes and rectal biopsies are the primary source of Tat-SL8 escape mutants during acute SIV_mac239_ infection

**Figure 3:**
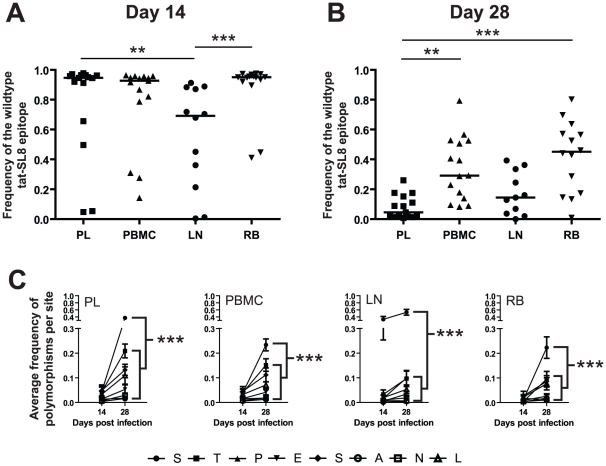
Escape in tat-SL8 was most evident in LN at day 14 post infection and variants generated there had spread to other tissues by day 28 post infection. (A–B) The level of tat-SL8 escape mutants expressed as the frequency of the wildtype sequence in plasma virus (PL), peripheral blood mononuclear cells (PBMC), lymph nodes (LN), and rectal biopsies (RB) at day 14 (A) and day 28 post infection (B). Significant differences were determined with the Kruskal-Wallis test with Dunn's Multiple Comparisons **: p<0.01, ***: p<0.001. (C) The average frequency of mutations per amino acid position in tat-SL8 across all animals is shown. 2-way ANOVA using the Bonferroni Correction for multiple comparisons; ***: p<0.001. In each case the frequency of mutants at the first position (“S”) at day 28 post infection is compared individually to that at the other 7 amino acid positions in the epitope. This comparison was only significant in LNs at day 14 post infection.

To investigate the kinetics of appearance and dissemination of escape mutants in SIV_mac239_ infection in different anatomic compartments, we next compared both the frequency and the character of Tat-SL8 CTL escape mutants in viral sequences derived from plasma virus and cell-associated viral DNA from peripheral blood mononuclear cells (PBMCs), lymph nodes (LNs) and intestinal mucosa that was sampled by rectal biopsies (RB). This analysis was performed on samples obtained at day 14 and day 28 post infection. While Tat-SL8 escape mutants were evident in all four examined tissues at day 14 post infection, LNs exhibited a significantly higher frequency of escape mutants than either plasma or RB (Figure 3A; p = 0.0022, Kruskal-Wallis with Dunn's multiple comparisons). Interestingly, the discrepancy in the level of escape between LNs and plasma virus may be seen as reflecting the fact that circulating virus at the peak of acute SIV infection originates primarily from other tissues. By day 28 post infection, plasma virus exhibited a greater level of Tat-SL8 escape than cell-associated virus sampled from either PBMCs or RBs (Figure 3B; p = 0.0001, Kruskal-Wallis with Dunn's test for multiple comparisons), but not from LNs. This finding suggests that plasma virus during the phase of post-peak decline in viremia is likely to originate primarily in lymphoid tissues.

Escape in Tat-SL8 can occur through multiple amino acid substitutions [14], [23], [24]. In order to further delineate the sources of actively replicating viruses that contain Tat-SL8 escape mutants during acute SIV_mac239_ infection of RMs, we next characterized the frequency distribution of distinct intra-epitopic Tat-SL8 escape mutations. We found that, in the majority of RMs, Tat-SL8 escape mutants sampled from LNs at day 14 post infection were dominated by a serine to proline amino acid substitution at the first position of the epitope, although amino acid substitutions at other positions were observed at much lower frequency (Figure S1A). This pattern of amino acid substitution observed in LNs appeared to be different from what we observed in the Tat-SL8 genotypes sampled from the other three examined tissues, in which there was no clearly dominant amino acid substitution (Figure S1A). In particular, the distribution of Tat-SL8 escape mutants in the plasma virus was much more similar to that of cell-associated viral DNA from PBMCs, with small, equivalent frequencies of mutation at the first, second, fifth and eighth positions, than that of either RBs or LNs. Of note, by day 28 post infection, the distribution of Tat-SL8 escape mutants had become more similar across all tissues (Figure S1B). However, while we observed an increase in the relative abundance of mutations at positions 2 through 8 in LNs, the serine to proline mutation at the first amino acid position of Tat-SL8 continued to be dominant (Figure S1B). In fact, this particular escape mutation became the most frequently sampled mutation in all tissues at day 28 post infection (Figure 3C; 2-way ANOVA with Bonferroni multiple comparison test, p<0.001), and continued to dominate viral populations through the latest time points sampled from all tissues (data not shown). The persistent high frequency of this escape mutation at the first position of Tat-SL8 suggests that anti-SIV immune responses during the acute infection in lymphoid tissues rather than at mucosal sites have a more lasting effect on the evolution of the SIV viral population.

Interestingly, three of the four RMs exhibiting relatively large numbers of escape mutants at day 14 post infection had been vaccinated with the MVA-SIV vector (Figure S1A), thus suggesting an early CTL-driven selection of these viral variants. Indeed, vaccinated RMs showed a non-significant trend towards increased frequencies of Tat-SL8 escape mutants at day 14 post infection compared to unvaccinated controls (p>0.05, Kruskal-Wallis with Dunn's test for multiple comparisons). However, vaccination did not preferentially increase the level of Tat-SL8 specific CD8+ T cells in LNs compared to PBMCs and RBs at days −28, 0, and 7 post infection (Figure S2). Taken together these observations argue against the possibility that the expansion of Tat-SL8 escape mutants in LNs was caused primarily by vaccine-induced prior expansion or redistribution of Tat-SL8-specific CD8 T cells to lymphoid tissues.

### The frequency of Tat-SL8 escape mutants at day 28 post infection is correlated with the magnitude of SIV-specific immune responses at day 14 post infection

**Figure 4:**
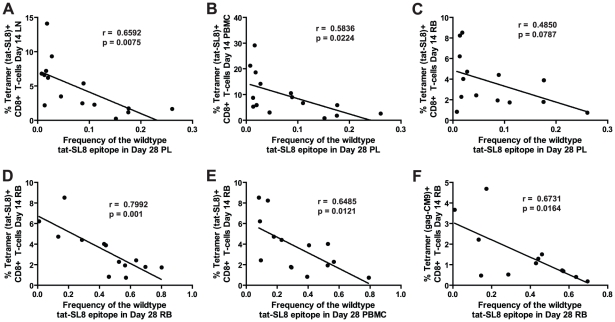
Anti-SIV immune responses in lymphoid tissues and rectal mucosa at day 14 post infection were significantly correlated with the level of tat-SL8 escape at day 28 post infection in the plasma virus and rectal mucosa, respectively. (A–B) The frequency of the wildtype tat-SL8 at day 28 post infection in plasma virus plotted against the percentage of tat-SL8-tetramer-positive CD8 T cells at day 14 post infection in lymph nodes (A) and PBMCs (B). (C–F) The frequency of wildtype tat-SL8 at day 28 post infection in RB (C,E) and PBMC (D,F) plotted against the percentage of tat-SL8-tetramer-positive (C–D) and gag-CM9-tetramer-positive (E–F) CD8 T cells in RB at day 14 post infection. Spearman's correlation was used to determine the degree of correlation in all comparisons.

The large amount of virological and immunological data collected during the course of SIV_mac239_ infection in the RMs included in this study allowed us to probe whether specific immune responses or virological outcomes (see Table S1) were associated with the levels of CTL escape mutants observed at days 14 and 28 post infection. We did not find any significant correlations between the levels of escape mutants in any tissue at day 14 post infection and the levels of SIV-specific CD8+ T cell-mediated immune responses as measured by tetramer staining at the same time point. Furthermore, none of the virological parameters measured at days 14 and 28 post infection correlated with the level of escape mutants present in any tissue at the same time points. However, we observed a clear correlation between immune responses at day 14 post infection and the level of Tat-SL8 escape present in several tissues at day 28 post infection (Figure 4). First, the frequency of viruses bearing the wildtype Tat-SL8 epitope among plasma viruses at day 28 post infection was significantly inversely correlated with the abundance of anti-Tat-SL8 CD8+ T cells at day 14 post infection in LNs (Figure 4A; Spearman's correlation, r = 0.6592, p = 0.0075) and PBMCs (Figure 4B; Spearman's correlation, r = 0.5836, p = 0.0224), but not RBs (Figure 4C; Spearman's correlation, r = 0.4850, p = 0.787), suggesting that LNs are the major anatomic sites where the anti-SIV CD8 T cell response is exerting immunological pressure at the peak of acute SIV_mac239_ infection. Second, the frequency of wildtype Tat-SL8 epitope in RBs and PBMCs at day 28 post infection was strongly correlated with levels of Tat-SL8 CD8+ T cells in RBs at day 14 post infection (Figure 4D–E; Spearman's correlation, RB: r = 0.7992, p = 0.001; PBMC: r = 0.6485, p = 0.0121), perhaps suggesting a local effect of mucosal SIV-specific CTL responses in determining the emergence of escape mutants. Third, the levels of Gag-CM9-specific CD8+ T cell responses in RBs at day 14 post infection also correlated strongly with the frequency of tat-SL8 escape mutants in RBs (Figure 4F; Spearman's correlation, r = 0.6731, p = 0.0164), but not PBMCs (data not shown) at day 28 post infection. Interestingly, despite the significant correlations between the frequency of Tat-SL8 escape and the magnitude of anti-SIV CD8 T cell responses in various tissues, the decline in CD4+ T cells in RB and PBMC did not correlate with the frequency of escape at 14 and 28 days post infection in any tissue (data not shown). Taken together these findings indicate that SIV-specific CTL responses at the peak of acute infection have a strong impact on the resultant viral population, with the pattern of observed circulating virus variants at day 28 post infection being shaped predominantly by LN-based immune responses.

### RMs that control virus replication harbor higher levels of proviruses bearing the wildtype Tat-SL8 in PBMC than non-controller animals

**Figure 5:**
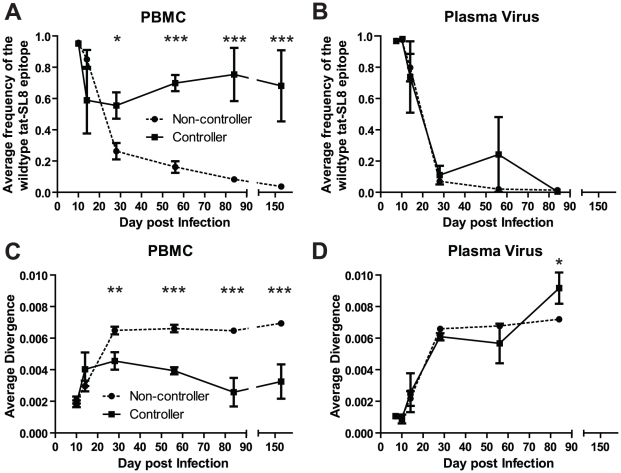
Control of SIVmac239 replication was associated with significantly higher levels of cell-associated viral DNA bearing the wildtype tat-SL8 sequence in PBMC but not in plasma viral RNA. (A–B) The average frequency of wildtype tat-SL8 in controllers and non-controllers was determined in PBMC (A) and plasma virus (B). (C–D) The average nucleotide sequence divergence from the wildtype sequence in the 220 nucleotide region surrounding tat-SL8 is shown in PBMC (C) and plasma virus (D). 2-way ANOVA using the Bonferroni Correction for multiple comparisons; *: p<0.05, **: p<0.01, ***: p<0.001.

Four SIV-infected RMs included in the current study (three vaccinated and one control) were able to spontaneously control virus replication to near undetectable levels shortly after the resolution of acute viremia (Table S1). These RMs exhibited a ∼1 log lower peak of acute viremia, yet similar levels of SIV-specific CD8+ T cell responses as the normal non-controller animals. To understand the contribution of CD8+ T cell escape to the control of SIV replication, we compared the prevalence of Tat-SL8 escape mutants and the diversity of virus sequences between the controller and non-controller groups of SIV-infected RMs. Control of SIV_mac239_ replication in RMs was associated with increased levels of viruses bearing the wildtype Tat-SL8 epitope among infected cells in PBMCs (Figure 5A; 2-way ANOVA using the Bonferroni Correction for multiple comparisons) while viruses circulating in the plasma were primarily escape mutants in both groups (Figure 5B). Similarly, viruses sampled from RBs in controllers at day 168 post infection were also composed primarily of viruses bearing the wildtype Tat-SL8 epitope (data not shown). It should be noted, however, that virus was amplifiable from day 168 RB samples of only two of the four controller. Finally, Tat-SL8 and the surrounding region more closely resembled wildtype SIV_mac239_ in viruses sampled at late time points from PBMC, but not plasma, of controllers than non-controllers (Figure 5C–D; 2-way ANOVA using the Bonferroni Correction for multiple comparisons). In the setting of low virus replication, as in our controller SIV-infected RMs, virus isolated from cellular samples may include a large representation of archival sequences as compared to plasma. As such, it is possible that the large frequency of wildtype viruses in the controllers is a consequence, rather than a cause, of the low virus replication.

## Discussion

Despite the presence of a strong antiviral immune response, HIV infection of humans and SIV infection of RMs usually results in a chronic and progressive immunodeficiency. The inability of anti-SIV immune responses to effectively control virus replication is at least partially due to the singular ability of this primate lentivirus to evade immune responses via a high rate of genetic mutation [13]. The epidemic circulation of extremely diverse viral subtypes and the immune-mediated selection of viral escape variants are two of the main problems that must be overcome by a successful candidate HIV vaccine [25]. In the setting of experimental SIV infection of non-human primates, the comparison of virus sequences obtained in different tissues might be used to determine the anatomic origin of these escape variants. We postulate that this analysis will help us define the anatomic compartments where immune responses have their greatest impact on virus replication and fitness. In addition, as plasma virus includes all viral variants produced in the body at any given time, a comparative kinetic analysis of the emergence of viral escape mutants in plasma vs. tissues could provide an indirect but robust measure of the extent of virus replication in the examined tissues. Identifying these anatomic sites of high viral replication and dominant immune pressure during the acute phase of HIV/SIV infection might help the design of a vaccine that can elicit adaptive immune responses capable of durable control of HIV replication.

In order to define these sites of virus replication and immune pressure during acute SIV_mac239_ infection of RMs, we investigated the kinetics and characteristics of escape in two well characterized Mamu-A*01-restricted CTL epitopes (Gag-CM9 and Tat-SL8). We sequenced the SIV genomic regions containing these epitopes in longitudinal archival samples of plasma virus and cell-associated viral DNA from PBMCs, LNs and RBs collected from a cohort of vaccinated and challenged RMs [22]. Ten of the rhesus macaques in this study had been vaccinated with MVA-derived vectors expressing the Gag-CM9 and Tat-SL8 epitopes, while five of the challenged RMs were unvaccinated. While our data do not rule out the possibility that other tissues (i.e., spleen, liver, etc) are significant contributors to plasma viremia and/or immune pressure, the unique aspect of this study is the direct comparison between plasma, LNs, PBMCs, and RBs.

As expected, Gag-CM9 escape mutants were observed in only two animals and after day 84 post infection, while the Tat-SL8 epitope acquired escape mutations very quickly during the acute phase infection. Interestingly, the comparative analysis of Tat-SL8 escape mutants revealed, at day 14 post infection, a higher frequency in LN as compared to other tissues. By day 28 post infection, Tat-SL8 escape mutants had become dominant in plasma virus, and were found at higher frequency in LNs as compared to RBs. Of note, at day 14 post infection virus escape mutants in LNs, but not other tissues, were overwhelmingly comprised of mutations at the first amino acid of the epitope. These mutants became dominant in all tissues by day 28 post infection. Taken together, these results suggest that, during acute SIV_mac239_ infection, lymph nodes are the main sites of virus replication and/or cellular immune pressure.

The large amount of virological and immunological data collected during the original vaccination and challenge study of these SIV-infected RMs [22] allowed us to more explicitly test hypotheses regarding the immunological processes and anatomic compartments that ultimately produced these viral escape mutants. By correlating immune responses and changes in viral dynamics with the level of escape in the examined tissues, we can determine not only the origin of escape mutants, but the immune responses responsible for their generation. We found that the level of Tat-SL8-specific immune responses in LNs, and to a lesser extent PBMCs, at day 14 post infection correlated strongly with the level of escape in plasma virus at day 28 post infection. On the other hand, SIV-specific immune responses in RBs at day 14 post infection did not correlate with the level of Tat-SL8 escape at day 28 in plasma virus. Therefore, LNs rather than mucosal tissues appear to be the tissue in which the strongest immune pressure shapes the genetic composition of the currently replicating virus population during the phase of post-peak decline of viremia in SIV-infected RMs.

In addition, we observed that, in controller SIV-infected RMs (but not in non-controller RMs), a large proportion of PBMC-derived proviruses during the chronic phase of infection exhibited the wildtype Tat-SL8 rather than the escaped epitope. This high frequency of wildtype virus in controller RMs may be the result of the early establishment of a wildtype latent viral reservoir, which, due to their low level of replication, appears over-represented as compared with the non-controller animals. Since the actively replicating virus (i.e., in plasma) contains very few, if any, viruses bearing the wildtype Tat-SL8, these wildtype PBMC-associated viruses are likely to be either latent or non-replication competent.

The observation that immune responses in LNs are more important for shaping post acute viral populations is somewhat surprising as the gut-associated lymphoid tissue has been implicated as the major source of virus replication during the acute phase of SIV infection [26], [27], [28], [29], [30]. One possibility is that, during the post-peak decline of viremia, the gut mucosa is a relatively isolated site of SIV replication, with slower migration of virions from the intestinal interstitial fluids into the systemic circulation as compared to LNs. Indeed, there is evidence for compartmentalization of HIV within the gut [31] suggesting that virus does not move freely within this tissue. An alternative possibility is that the level of immunological pressure, rather than the absolute level of virus production, is responsible for the directional appearance of CTL escape mutants from LNs to mucosal tissues. This concept fits with the fact that during acute SIV infection LNs are the site of antigen-specific CD8+ T cell expansion from the naïve pool, with virus-infected cells encountering anti-SIV CD8+ T cells more frequently than in the intestinal lamina propria. These data are also consistent with the possibility that the gut is not the main source of virus replication during acute SIV infection, as recently suggested in a modeling study [32]. However, we could not rule out the possibility that the mutations in Tat simply provide a selective advantage to viruses in lymphoid tissue (or less of a disadvantage relative to viruses replicating in gut mucosal tissues) independently of their effects on immune recognition.

While our study agrees with the numerous published articles that have characterized immune escape in SIV_mac239_ infected RMs [14], [15], [17], [23], [33], [34], [35], [36], [37], in almost all of these studies, CTL escape mutants were assessed only in plasma virus. Our study is unique in that we examined virus in multiple longitudinal tissue samples (PBMC, LNs, and RBs) in addition to the plasma virus. We are in fact aware of only a single study that examined SIV_mac239_ escape mutants in multiple tissues [38], but this study focused only on Gag-CM9 escape measured at a single time point during the chronic infection. It will be important to determine whether the pattern of escape seen in Tat-SL8 during the acute infection is a general phenomenon for CTL epitopes that escape during the acute phase of infection with little or no measurable effects on viral fitness. In addition it will be informative to characterize the anatomic distribution of CTL epitopes that escape during the chronic phase of SIV infection (e.g. Gag-CM9) by designing new studies in which tissue samples are collected accordingly. Furthermore, future studies should address whether the cell-associated viral sequences sampled from relatively small biopsies of the rectal mucosa are representative of the gut as a whole. While this caveat applies to most studies of gut-associated lymphoid tissue in the setting of SIV infection, it is possible that our current results are biased by the limited number of samples available for virus sequencing.

It is unclear whether and to what extent the particular pattern of CTL escape observed in our cohort of SIV-infected RMs represents a generalized phenomenon or, alternatively, is the result of this specific experimental design (i.e., intravenous high titer infection), which does not mimic the typical route and circumstances surrounding HIV infection of humans. Furthermore, the route of infection may influence the anatomic pattern of emergence of CTL escape mutants. For example, a rectal challenge model might result in the early seeding of local draining lymph nodes with virus whereas an intra-venous challenge could possibly disseminate virus more efficiently to systemic lymphoid tissues throughout the body. Future studies in which RMs are infected with low dose intra-rectal or intra-vaginal challenge will help determine how the route and dose of infection influences the early kinetics of CTL escape. Additionally, while the MVA vaccination regime in this study did not protect from SIV disease progression, it would be interesting to determine whether more successful vaccines (i.e., rhesus CMV [39] and AdHu26 [40]) alters the dissemination of viral escape variants and/or the pattern of immune responses during the acute phase of SIV infections.

## Materials and Methods

### Ethics statement

These studies were carried out in strict accordance with the recommendations in the Guide for the Care and Use of Laboratory Animals of the National Institutes of Health, and were approved by the Emory University (AWA# A3180-01) and University of Pennsylvania (AWA# A3079-01) Institutional Animal Care and Use Committees. All animals were anesthetized prior to the performance of any procedure, and proper steps were taken to ensure the welfare and to minimize the suffering of all animals in these studies.

### Immunization and SIV_mac239_ infection of RMs

The design of the SIV immunization and challenge study in RMs that has been used as a source of samples for the current manuscript are published elsewhere [22]. Briefly, ten RMs were divided in two groups of five animal and immunized three times with either one of two MVA vaccine vectors (i.e., wildtype and a genetically modified MVA in which the *udg* gene was deleted) expressing the SIVmac239 *gag* and *tat* genes. One year after the initial immunization, all ten animals as well as five unvaccinated control RMs were inoculated i.v. with 10,000 TCID50 of SIV_mac239_. Throughout the immunization and challenge phase plasma, peripheral blood mononuclear cells (PBMC), lymph node biopsies (LN) and biopsies of the rectal mucosa (RB) were collected for virological and immunological analysis (see Table S1). The MHC-class I genotypes of all fifteen RMs were determined courtesy of Dr. David Watkins. Of note, exclusion of the only MamuB*08+ and MamuB*17+ animal did not change the result of our analyses.

### Tetramer staining

PBMC were collected by gradient centrifugation. LN and RB were processed fresh as previously described. The levels of Tat- and Gag- specific CD8 T cells were assessed via staining with Streptavidin-APC conjugated class I MHC tetramers folded with the Tat_28-35_-SL8 (STPESANL) or Gag_181-189_-CM9 (CTPYDINQM) peptide epitopes according to standard procedures.

### Tissue processing and viral sequence amplification

Genomic DNA was purified from thawed PBMC samples using the QIAamp DNA blood mini kit (Qiagen; Valencia, CA), and from whole Streck-fixed, paraffin embedded RBs or from 50 µM slices of formalin-fixed, paraffin embedded LNs using the QIAamp DNA FFPE tissue kit (Qiagen; Valencia, CA). Viral RNA was extracted from plasma samples using the Qiagen's Viral RNA mini kit (Valencia, CA). Viral cDNA was reverse transcribed using Invitrogen's SuperScript III and primers specific for sequences upstream of the *tat* (Tat-RT3: 5′-TGGGGATAATTTTACACAAGGC-3′) or *gag* (Gag-RT2: 5′-AGCTTGCAATCTGGGTTAGC-3′) amplicons.

Viral sequences were amplified from purified genomic DNA or viral cDNA in a nested two step PCR. The thermal cycler program for the first round was: 94°C for 2:00; cycle 94°C for 0:30, 55°C for 0:30, and 68°C for 1:00, 35 times; and then a final 68°C for 7:00 before cooling to 4°C. After the tenth cycle, the extension step (68°C) is extended by 5 seconds every cycle to account for the degrading polymerase. The thermal cycler program for the second round was: 94°C for 2:00; cycle 94°C for 0:30, 53°C for 0:30, and 68°C for 1:00, 35 times; and then a final 68°C for 7:00 before cooling to 4°C. The first round primers for *tat* were Tat-F1 (5′-GATGAATGGGTAGTGGAGGTTCTGG-3′) and Tat-R2 (5′-CCCAAGTATCCCTATTCTTGGTTGCAC-3′), and the first round primers for *gag* were Gag-F1 (5′-GAGACACCTAGTGGTGGAAACAGG-3′) and Gag-R2 (5′-GCTCTGAAATGGCTCTTTTGGCCC-3′). The design of the second round primers involved the incorporation of Roche's 454 Adaptor sequences and an animal-specific, 4 nucleotide barcode that allows identification of individual animals in the pooled sequencing runs. The barcode key can be found in Table S1, and their position is indicated by a lowercase ‘b’ in the following primer sequences. Second round primers for *tat* had sequences similar to those previously published [15] and were Tat-F3 (5′-GCCTTGCCAGCCCGCTCAGbbbbTGATCCTCGCTTGCTAACTG-3′) and Tat-R3 (5′-GCCTCCCTCGCGCCATCAGAGCAAGATGGCGATAAGCAG-3′), and the second round primers for *gag* were Gag-F3 (5′-GCCTTGCCAGCCCGCTCAGbbbbCACCATCTAGCGGCAGAGGAGG-3′) and Gag-R3 (5′-GCCTCCCTCGCGCCATCAGACCCCAGTTGAATCCATCTCCTG-3′). After amplification, each amplicon was gel purified using the QIAquick gel extraction kit (Qiagen, Valencia, CA). The amplicons were then quantified on a NanoDrop (Company), and mixed at equimolar concentrations within tissues and time points. Massively parallel pyrosequencing was performed by the University of Pennsylvania, Department of Genetics Sequencing Facility on a Roche 454 Genome Sequencer FLX (Branford, CT).

### Sequence and statistical analysis

Raw 454 sequencing reads will be available in the NCBI Sequence Read Archive under accession number SRA027346.1. Sequences were subjected to several steps of quality control [41], [42]. First, sequences that contained ambiguous nucleotides and those that did not meet the minimum length requirements (tat: 220 nucleotides; gag: 243 nucleotides) were excluded. Next, each read was aligned individually to the SIVmac239 wildtype sequence using ClustalW and barcodes were identified. Reads where the barcode could not be identified were excluded from the analysis. Several common insertions and deletions (indels) that resembled common 454 sequencing artifacts were then identified and repaired using the wildtype SIVmac239 sequence as a template. Sequences containing multiple indels and low frequency sequencing artifacts were excluded. All sequence manipulations and analyses were performed and implemented in a suite of scripts written in Python. Statistical analyses were all performed in GraphPad Prism.

## Supporting Information

Figure S1 The distribution of mutated sites in Tat-SL8 differs between between tissues. The frequency of mutants at each amino acid position in the tat-SL8 epitope (underlined) and the 5 upstream and downstream positions are shown at day 14 (A) and day 28 post infection (B) in all four tissues. Animals are grouped by vaccination (MVAΔudg-SIV, MVA-SIV, and unvaccinated).
(PDF)

Figure S2 The frequency of Tat-SL8-specific CD8+ T cells does not differ between PBMCs, LNs, and RBs at day -28, day 7, and day 14 post infection. The frequency of Tat-SL8-specific CD8+ T cells in PBMCs, LNs, and RBs is shown for vaccinated and unvaccinated animals at (A) day -28, (B) day 7, and (C) day 14 post infection. The line represents the average for either vaccinated (open circle) or unvaccinated (filled square) RMs. No differences were significantly different (2-way ANOVA, p>0.05).
(PDF)

Table S1 Summary of virological and immunological parameters. Viral loads are measured in viral RNA copies per mL of plasma. All immunological parameters were measured via flow cytometry.
(XLS)
