# Supplementary figures and images for: Viral CTL Escape Mutants Are Generated in Lymph Nodes and Subsequently Become Fixed in Plasma and Rectal Mucosa during Acute SIV Infection of Macaques
Source: PLoS Pathog. 2011 May 19;7(5):e1002048. doi: 10.1371/journal.ppat.1002048 (PMC3098234; doi:10.1371/journal.ppat.1002048)

**A****Day 14**

Frequency of polymorphisms

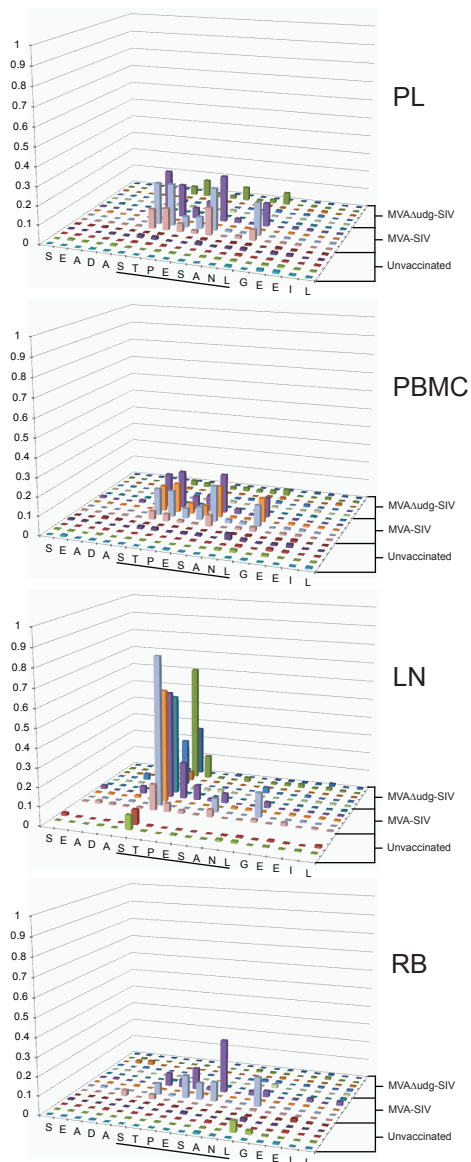**B****Day 28**

Frequency of polymorphisms

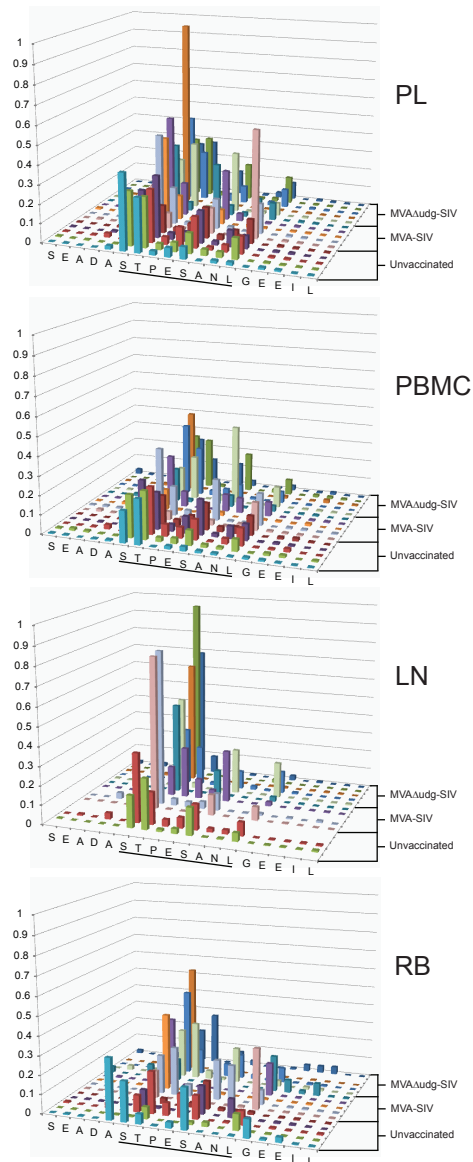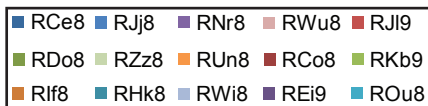

Supplement: Figure S1 — The distribution of mutated sites in Tat-SL8 differs between between tissues. The frequency of mutants at each amino acid position in the tat-SL8 epitope (underlined) and the 5 upstream and downstream positions are shown at day 14 (A) and day 28 post infection (B) in all four tissues. Animals are grouped by vaccination (MVAΔudg-SIV, MVA-SIV, and unvaccinated). (PDF) [file ppat.1002048.s001.pdf]

# Supplemental Figure 1

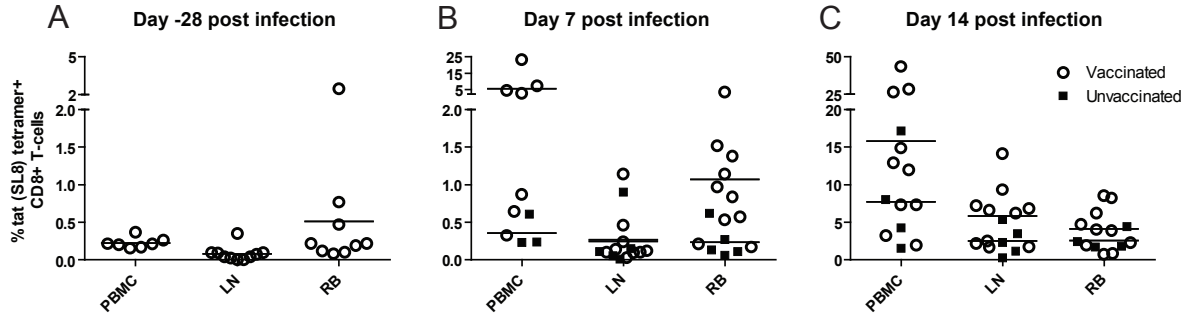

Supplement: Figure S2 — The frequency of Tat-SL8-specific CD8+ T cells does not differ between PBMCs, LNs, and RBs at day -28, day 7, and day 14 post infection. The frequency of Tat-SL8-specific CD8+ T cells in PBMCs, LNs, and RBs is shown for vaccinated and unvaccinated animals at (A) day -28, (B) day 7, and (C) day 14 post infection. The line represents the average for either vaccinated (open circle) or unvaccinated (filled square) RMs. No differences were significantly different (2-way ANOVA, p>0.05). (PDF) [file ppat.1002048.s002.pdf]
